# A new intuitionistic fuzzy rule-based decision-making system for an operating system process scheduler

**DOI:** 10.1186/s40064-016-3216-z

**Published:** 2016-09-13

**Authors:** Muhammad Arif Butt, Muhammad Akram

**Affiliations:** 1 Punjab University College of Information Technology, University of the Punjab, Old Campus, Lahore, 54000 Pakistan; 2 Department of Mathematics, University of the Punjab, New Campus, Lahore, Pakistan

**Keywords:** Operating system, CPU scheduler, Scheduling algorithms, Fuzzy sets, Intuitionistic fuzzy logic, Intuitionistic fuzzy logic controller, Defuzzification

## Abstract

We present a new intuitionistic fuzzy rule-based decision-making system based on intuitionistic fuzzy sets for a process scheduler of a batch operating system. Our proposed intuitionistic fuzzy scheduling algorithm, inputs the nice value and burst time of all available processes in the ready queue, intuitionistically fuzzify the input values, triggers appropriate rules of our intuitionistic fuzzy inference engine and finally calculates the dynamic priority (dp) of all the processes in the ready queue. Once the dp of every process is calculated the ready queue is sorted in decreasing order of dp of every process. The process with maximum dp value is sent to the central processing unit for execution. Finally, we show complete working of our algorithm on two different data sets and give comparisons with some standard non-preemptive process schedulers.

## Background

To tame the wild hardware inside every computer system we need a specialized software called operating system (OS). As the name suggests an operating system is used to operate the hardware and provides services to different application programs. While doing so the main task of an OS is providing an interface that is easy to use and at the same time makes the best utilization of the underlying hardware. In todays world of computing there are multiple processes/threads executing concurrently and requesting for different services from the OS. The operating system has to deal efficiently with these finite resources and competing demands. To achieve this goal the operating system has to allocate and deallocate the hardware resources among various processes in an orderly, fair, and secure manner. Some of these hardware resources are space multiplexed and some are time multiplexed among the processes (Butt and Akram 2015). For example memory is one of the important resources that is space multiplexed among the processes. Every process along with its code, data and various control structures need to reside inside the main memory to execute properly. Memory management unit, normally called the MMU is the component of the operating system kernel which implements different space management algorithms to efficiently utilize the main memory. Similarly another important hardware resource that is time multiplexed among the processes residing in memory is the central processing unit (CPU). The responsibility of efficiently utilizing this important hardware resource and time multiplexing it among various processes lies on a kernel component called CPU scheduler. The CPU scheduler also called the process scheduler gets activated on every process switch. Once activated, the CPU scheduler selects a process from ready queue based on multiple attributes and also decides the duration for which this process will execute on the central processing unit.

The scheduling algorithm of UNIX operating system uses a simple formula to assign a priority value to every ready to run process. This per process attribute is calculated after every second and thus causes process priorities to change dynamically. When it is time for scheduling, the CPU is given to the process with the smallest priority number (Kerrisk 2010).

$$Priority\;value = Base\;priority + Nice\;value + (Recent\;CPU\;usage)/2$$

In the above formula, base priority is an integer usually having value of 60, while the default value of nice for a normal process is 0 and its range can be from −20 to 19. A user can change the priority of a process by changing its nice value using the UNIX *nice(1)* and *renice(1)* commands as shown below:

- *$ nice -val command [args]*
- *$ renice val pid*

By increasing the nice value (Butt and Akram 2015) of a process the overall priority of a process decreases and vice versa. When a system tries to run too many high priority jobs at the same time, computer response time deteriorates. The UNIX *renice(1)* command is used to adjust scheduling priorities to avoid this problem. Scheduler in Linux kernel 2.6 onwards, is the piece of kernel code that inputs a list of runnable threads and decides which thread will be executed by the CPU next based on per process parameters like, scheduling policy, nice value, dynamic priority and CPU affinity (Kerrisk 2010).

Shortest job first (SJF), first-come-first-served (FCFS) are non-preemptive, while Round-robin (RR), shortest remaining time first (SRTF), and multi-level feed back queue are some of the preemptive versions of CPU scheudling algorithms (Galvin et al. 2013). CPU scheduling is a multiple-object decision making process with multiple factors like response, waiting and turn-around times, CPU utilization, and throughput (Butt and Akram 2015; Lin et al. 2007; Xu and Cai 2012).

Real world problems are often complex where information obtained are not always complete. In many decision making scenarios we have more than two options available and many a times complete information about those options are not available. These cases cannot be modeled using simple set theory and Boolean Logic. To handle such situations we cnormally use fuzzy set theory (Zadeh 1965) and fuzzy logic (Zadeh 1975). In spite of its vast applications, fuzzy sets suffer with the limitation of uncertainity element due to non-availability of complete information. Among extensions of fuzzy sets, Atanassovs intuitionistic fuzzy sets (IFSs; Atanassov 1986) provide an intuitive framework to deal vagueness from imprecise information by taking into account non-membership values in addition to membership values. More recently, fuzzy logic and its derivatives like Intuitionistic fuzzy logic have find its place in the areas of expert systems, robotics, computer networks, social sciences, management sciences, life sciences, and image processing. Early work in fuzzy decision making was motivated by the desire to mimic the control actions of an experienced human operator and to obtain smooth interpolation between discrete outputs that would normally be obtained (Atanassov 2015; Akram et al. 2014a, b; Ashraf et al. 2014a). A fuzzy controller is normally comprised of a fuzzification module, a rule base, an inference engine and a defuzzification system. Several fuzzy controller have been developed using fuzzy logic and fuzzy set theory (Liu et al. 2013; Shen et al. 2013; Boldbaatar and Lin 2015).

Applying different fuzzy models for decision-making problems inside an operating system is a hot area of research these days. CPU scheduler is one of the important kernel component that is responsible for selection of a process, the time quantum the selected process should execute on CPU and last but not the least, how frequently the OS should invoke the scheduler. Varshney and Akhtar (2012) gave the idea of achieving an ideal CPU time slice using fuzzy logic. The achieved time slice value is neither too large making it behave like FCFS nor too small and increasing the context switch overhead manifold. For multiprocessor systems, a fuzzy CPU scheduling system has been proposed by Hamzeh et al. (2007). Lim and Cho (2007) after differentiating bach, interactive and real-time processes have also proposed an intelligent fuzzy CPU scheduling system. They have used the CPU ticks a process executes and the sequence of system calls a process makes to differentiate between the three flavors of processes. A lot of research has been carried out in designing of fuzzy decision-making systems which takes input parameters like processes’s response ratio, priority values, waiting and remaining burst time. The fuzzification of these input parameters and designing of inference engines that mimic behavior of human experts, their results have shown improved average waiting and turn around times (Ajmani and Sethi 2013; Alam et al. 2011; Behera et al. 2012).

The authors already proposed a novel fuzzy decision-making system for a multi-tasking CPU scheduler (Butt and Akram 2015) and have compared their improved results with Behra’s IFCS (Behera et al. 2012) and Ajmain’s PFCS (Ajmani and Sethi 2013). This is an extension of the same algorithm but using intuitionistic fuzzification techniques for a batch operating system. By applying intuitionistic fuzzy (IF) logic in the decision-making process for the selection of next runnable thread, we have incoorporated the features of shortest job first as well as priority based CPU scheduler. The nice value and the burst time are the two per process parameters, which are fuzzified intuitionistically. These intuitionistically fuzzified input parameters are given to the IF inference engine composed of nine rules. The IF inference engine fire the rules in the rule base and computes dynamic priority (dp). This dynamic priority (dp) is then defuzzified using TSK formula (Leekwijck and Kerre 1999). This procedure is repeated for every process in the run-queue and later the queue is sorted by the priority attribute of each process in decreasing order. When the scheduling decision is to be made, process at the head of the run-queue is selected for execution on the CPU.

Our paper has following sections. Main components of our proposed system and its algorithm are discussed in “Basic structure and algorithm” section. The intuitionistic fuzzifier, rule base and defuzzification techniques are discussed in “Components of IFS” section. The results generated using our proposed system are shown, discussed and compared in “Results and discussion” section. Finally we have concluded and given future research directions. We have used standard definitions and terminologies in this paper. For other notations, terminologies and applications not mentioned in the paper, the readers are referred to Akram et al. (2013), Ashraf et al. (2014b), Hsu (2015), Parvathi et al. (2013) and Xu and Liao (2015).

## Basic structure and algorithm

**Fig. 1:**
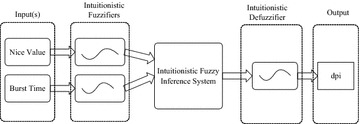
IFDMS for process scheduler

Figure 1 describes the basic structure of our proposed intuitionistic fuzzy decision-making system (IFDMS). The two inputs of our decision-making system are the per process attributes, namely, burst time and nice value. These two attributes of all the processes in the run-queue are intuitionistically fuzzified. These two input values of each process then triggers the rules in the rule base and finally we get the defuzzified dynamic priority (dp) for each process. The run-queue is sorted according to the decreasing value of dynamic priority and when there is a scheduling decision is to be made the process at the head of the run-queue is selected and given to the CPU for execution.

### Algorithm

1. Begin
2. IFIS := Create intuitionistic fuzzy inference system
3. Define linguistic values of input variables; *nice value* and *burst time*
4. Define linguistic values of output variable *dynamic priority (dp)*
5. Initialize Process Control Block of each process
6. nice_value := Read nice value from PCB of process
7. burst_time := Read burst time value from PCB of process
8. [Low Medium High] := Find degree of MF for nice_value
9. [NLow NMedium NHigh] := Find degree of NMF for nice_value
10. [Low Medium High] := Find degree of MF for burst_time
11. [NLow NMedium NHigh] := Find degree of NMF for burst_time
12. Trigger appropriate rules in the intuitionistic fuzzy (IF) rule base to get degree of truth for dynamic priority of process
13. Apply defuzzification formulas to get crisp value of dynamic priority
14. On arrival of every new process go to step step 6
15. Sort the queue of processes
16. Do the selection from head of run-queue
17. On termination of an executing process, go to step 16
18. End

## Components of IFS

Our proposed IFIS uses the Mamdani-Larsen inference method (Mamdani 1974). The three major components are the intuitionistic fuzzifier, intuitionistic fuzzy inference engine (IFIE) and the defuzzifier.

### Intuitionistic fuzzifier

**Fig. 2:**
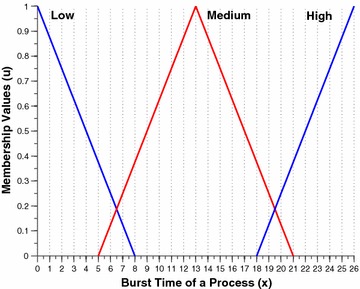
MF for burst time

**Fig. 3:**
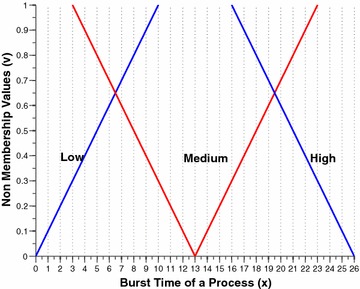
NMF for burst time

**Fig. 4:**
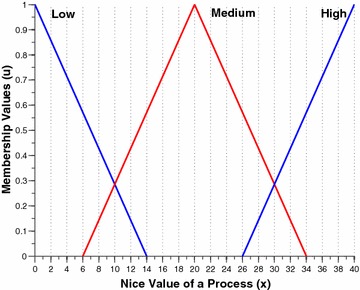
MF for nice value

**Fig. 5:**
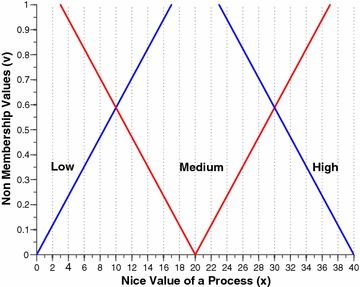
NMF for nice value

**Fig. 6:**
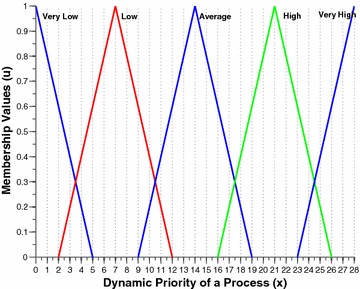
MF for dynamic priority

**Fig. 7:**
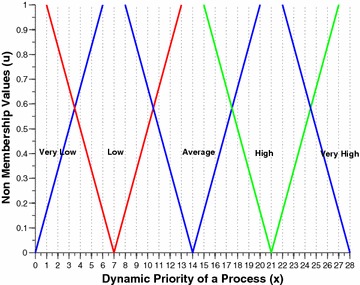
NMF for dynamic priority

The two per process attributes, namely the nice value and the burst time are intuitionistically fuzzified by this component. Figures 2 and 3 shows the membership funciton (MF) and non-membership function (NMF) for burst time of a process respectively. Similarly, Figs. 4 and 5 shows the MF and NMF for nice value of a process respectively. The output variable of our intuitionistic decision making system is the dynamic priority (dp) of a process. Figures 6 and 7 shows the MF and NMF for dynamic priority (dp) of a process respectively.

$$\begin{aligned} \mu _\mathrm{{low}}(x)&= {\left\{ \begin{array}{ll} \frac{8-x}{8-0} &{} \quad \text {if }x \in [0, 8],\\ 0 &\quad \text {else}, \end{array}\right. }\qquad \quad \nu _\mathrm{{low}}(x) = {\left\{ \begin{array}{ll} \frac{x-0}{10-0} &\quad \text {if }x \in [0, 10],\\ 1 &\quad \text {else}, \end{array}\right. }\\ \mu _\mathrm{{medium}}(x)&= {\left\{ \begin{array}{ll} \frac{x-5}{13-5} &\quad \text {if }x \in [5, 13],\\ \frac{21-x}{21-13} &\quad \text {if }x \in [13, 21], \\ 0 &\quad \text {else}, \end{array}\right. }\qquad \nu _\mathrm{{medium}}(x) = {\left\{ \begin{array}{ll} \frac{13-x}{13-3} &\quad \text {if }x \in [3, 13],\\ \frac{x-13}{23-13} &\quad \text {if }x \in [13, 23], \\ 1 &\quad \text {else}, \end{array}\right. }\\ \mu _\mathrm{{high}}(x)&= {\left\{ \begin{array}{ll} \frac{x-18}{26-18} &\quad \text {if }x \in [16, 26],\\ 1 &\quad \text {else}. \end{array}\right. }\qquad \nu _\mathrm{{high}}(x) = {\left\{ \begin{array}{ll} \frac{26-x}{26-16} &\quad \text {if }x \in [16, 26],\\ 0 &\quad \text {else}. \end{array}\right. } \end{aligned}$$

$$\begin{aligned} \mu _\mathrm{{low}}(x)&= {\left\{ \begin{array}{ll} \frac{14-x}{14-0} &\quad \text {if }x \in [0, 14],\\ 0 &\quad \text {else}, \end{array}\right. }\quad \quad \quad \nu _\mathrm{{low}}(x) = {\left\{ \begin{array}{ll} \frac{x-0}{17-0} &\quad \text {if }x \in [0, 17],\\ 1 &\quad \text {else}, \end{array}\right. }\\ \mu _\mathrm{{medium}}(x)&= {\left\{ \begin{array}{ll} \frac{x-6}{20-6} &\quad \text {if }x \in [6, 20],\\ \frac{34-x}{34-20} &\quad \text {if }x \in [20, 34], \\ 0 &\quad \text {else}, \end{array}\right. }\quad \quad \quad \nu _\mathrm{{medium}}(x) = {\left\{ \begin{array}{ll} \frac{20-x}{20-3} &\quad \text {if }x \in [3, 20],\\ \frac{x-20}{37-20} &\quad \text {if }x \in [20, 37], \\ 1 &\quad \text {else}, \end{array}\right. }\\ \mu _\mathrm{{high}}(x)&= {\left\{ \begin{array}{ll} \frac{x-26}{40-26} &\quad \text {if }x \in [26, 40],\\ 1 &\quad \text {else}. \end{array}\right. }\quad \quad \quad \nu _\mathrm{{high}}(x) = {\left\{ \begin{array}{ll} \frac{40-x}{40-23} &\quad \text {if }x \in [23, 40],\\ 0 &\quad \text {else}. \end{array}\right. } \end{aligned}$$

$$\begin{aligned} \mu _\mathrm{{v.low}}(x)&= {\left\{ \begin{array}{ll} \frac{5-x}{5-0} &\quad \text {if }x \in [0, 5],\\ 0 &\quad \text {else}, \end{array}\right. }\quad \quad \quad \nu _\mathrm{{v.low}}(x) = {\left\{ \begin{array}{ll} \frac{x - 0}{6-0} &\quad \text {if }x \in [0, 6],\\ 1 &\quad \text {else}, \end{array}\right. }\\ \mu _\mathrm{{low}}(x)&= {\left\{ \begin{array}{ll} \frac{x-2}{7-2} &\quad \text {if }x \in [2, 7],\\ \frac{12-x}{12-7} &\quad \text {if }x \in [7, 12], \\ 0 &\quad \text {else}, \end{array}\right. }\quad \quad \quad \nu _\mathrm{{low}}(x) = {\left\{ \begin{array}{ll} \frac{7-x}{7-1} &\quad \text {if }x \in [1, 7],\\ \frac{x-7}{13-7} &\quad \text {if }x \in [7, 13], \\ 1 &\quad \text {else}, \end{array}\right. }\\ \mu _\mathrm{{avg}}(x)&= {\left\{ \begin{array}{ll} \frac{x-9}{14-9} &\quad \text {if }x \in [9, 14],\\ \frac{19-x}{19-14} &\quad \text {if }x \in [14, 19], \\ 0 &\quad \text {else}, \end{array}\right. }\quad \quad \quad \nu _\mathrm{{avg}}(x) = {\left\{ \begin{array}{ll} \frac{14 - x}{14-8} &\quad \text {if }x \in [8, 14],\\ \frac{x - 14}{20-14} &\quad \text {if }x \in [14, 20], \\ 1 &\quad \text {else}, \end{array}\right. }\\ \mu _\mathrm{{high}}(x)&= {\left\{ \begin{array}{ll} \frac{x-16}{21-16} &\quad \text {if }x \in [16, 21],\\ \frac{26-x}{26-21} &\quad \text {if }x \in [21, 26], \\ 0 &\quad \text {else}, \end{array}\right. }\quad \quad \quad \nu _\mathrm{{high}}(x) = {\left\{ \begin{array}{ll} \frac{21 - x}{21-15} &\quad \text {if }x \in [15, 21],\\ \frac{x - 21}{27-21} &\quad \text {if }x \in [21, 27], \\ 1 &\quad \text {else}, \end{array}\right. }\\ \mu _\mathrm{{v.high}}(x)&= {\left\{ \begin{array}{ll} \frac{x-23}{28-23} &\quad \text {if }x \in [23, 28],\\ 0 &\quad \text {else}. \end{array}\right. }\quad \quad \quad \nu _\mathrm{{v.high}}(x) = {\left\{ \begin{array}{ll} \frac{28 - x}{28-22} &\quad \text {if }x \in [22, 28],\\ 1 &\quad \text {else}. \end{array}\right. } \end{aligned}$$

### Fuzzy inference engine

This component is the actual brain as the logic of our algorithm resides here. The reasoning is applied using IF-THEN type fuzzy rules. These IF-THEN rules formulate the conditional statements that comprise fuzzy logic. The inference engine triggers the appropriate IF-THEN rules in the knowledge base using Mamdani-Larsen inference method (Mamdani 1974). There are nine rules that are used for fuzzy inference:

1. if $\langle b\_time \; is \; low \rangle$ and $\langle nice \; is \; low \rangle$ then $\langle dp \; is \; veryhi \rangle$
2. if $\langle b\_time \; is \; low \rangle$ and $\langle nice \; is \; medium \rangle$ then $\langle dp \; is \; hi \rangle$
3. if $\langle b\_time \; is \; low \rangle$ and $\langle nice \; is \; hi \rangle$ then $\langle dp \; is \; hi \rangle$
4. if $\langle b\_time \; is \; medium \rangle$ and $\langle nice \; is \; low \rangle$ then $\langle dp \; is \; avg \rangle$
5. if $\langle b\_time \; is \; medium \rangle$ and $\langle nice \; is \; medium \rangle$ then $\langle dp \; is \; avg \rangle$
6. if $\langle b\_time \; is \; medium \rangle$ and $\langle nice \; is \; hi \rangle$ then $\langle dp \; is \; low \rangle$
7. if $\langle b\_time \; is \; hi \rangle$ and $\langle nice \; is \; low \rangle$ then $\langle dp \; is \; low \rangle$
8. if $\langle b\_time \; is \; hi \rangle$ and $\langle nice \; is \; medium \rangle$ then $\langle dp \; is \; verylow \rangle$
9. if $\langle b\_time \; is \; hi \rangle$ and $\langle nice \; is \; hi \rangle$ then $\langle dp \; is \; verylow \rangle$

### Defuzzifier

The defuzzifier component takes fuzzy dynamic priority (dp) of each process and defuzzify it to crisp dp. There are several techniques in the literature through which we can perform defuzzification. We apply Takagi Sugani formula (Leekwijck and Kerre 1999) for defuzzification. Takagi Sugani’s formula is:

$$\begin{aligned} x = \frac{ \sum _{j=1}^M x^{j} ((1 - \pi _{A^{j}}) \mu _{A^{j}} + \mu _{A^{j}} \pi _{A^{j}})}{ \sum _{j=1}^M ((1 - \pi _{A^{j}}) \mu _{A^{j}} + \mu _{A^{j}} \pi _{A^{j}})}, \end{aligned}$$

where

$$\begin{aligned} \mu _{A^{j}}= & {} \wedge _{i=1}^n \mu _{A_{i}^{j}}(x)\\ \nu _{A^{j}}= & {} \bigvee _{i=1}^n \nu _{A_{i}^{j}}(x)\\ \pi _{A^{j}}= & {} 1-\mu _{A^{j}}-\nu _{A^{j}} \end{aligned}$$

## Results and discussion

To generate the results we have used Process Scheduling Simulation, Analyzer and Visualization (PSSAV) tool for simulation of shortest job first (SJF) and non-preemptive priority based algorithm. PSSAV is publically available under code license GNU GPL v2. To generate the results of our own algorithm, a simulation is written using Matlab R2014a(8.3.0.532). The working to generate the results for our intuitionistic fuzzy decision-making system (IFDMS) for non-preemptive CPU scheduling algorithm is shown below. We have generated and compared the results using two different case studies.

The results of our two case studies are shown in Tables 7 and 8. The results of our intuitionitic fuzzy process scheduler are almost the same as the best known non-preemptive scheduling algorithm, i.e., shortest job first (SJF). The added advantange of using our algorithm is that we can further increase/decrease the priority of a process by decreasing/increasing its nice value respectively. The same cannot be done using SJF algorithm. So our proposed algorithm not only combines the plus points of conventional shortest job first (SJF) and priority based algorithm, but further gives reduced waiting times by adding the intuitionistic fuzzy modeling technique.

### Data set 1

The input for data set-1 is given in Table 1. These per process values are taken from author’s previous paper (Butt and Akram 2015). The recent cpu usage of each process is not computed here, as this input is not required for scheduling algorithms of batch operating systems. The first column shows the five processes under consideration, with their burst times (BT) and nice values (NV) shown against them.

**Table 1:** Data set-1

PID	BT	NV
P1	3	2
P2	6	7
P3	4	5
P4	5	6
P5	2	1

Table 1 shows that there are five processes with different burst times and nice values. We need to compute the dynamic priority of all the five processes. The detailed working for computing the dynamic priority of these processes is shown below.

#### Calculating dynamic priority of process P1

The first step is to intuitionistically fuzzify the crisp inputs, which can be obtained from the membership function (MF) and non-membership function (NMF) of burst time and nice value from Figs. 2, 3, 4 and 5 respectively. The fuzzified membership and non-membership values of burst time (3) and nice value (2) for process P1 are shown below:

$$\begin{aligned} \mu _\mathrm{{B.T}}&= \{0.625, 0, 0\} ; \nu _\mathrm{{B.T}} = \{0.3, 1, 1\} \\ \mu _\mathrm{{N.V}} &= \{0.857, 0, 0\} ; \nu _\mathrm{{N.V}} = \{0.118, 1, 1\} \end{aligned}$$

After intuitionistically fuzzifying the two input parameters the rules of inference engine are triggered. In case of membership rule1 is triggered: $low \wedge low = 0.625 \wedge 0.857 = 0.625$. In case of non-membership rule1 is triggered: $low \vee low = 0.3 \vee 0.118 = 0.3$. Finally, the membership and non-membership values of the output variable dynamic priority for process P1 are shown below:

$$\begin{aligned} \mu _\mathrm{{dp}} = \{0, 0, 0, 0, 0.625\} ; \nu _\mathrm{{dp}} = \{1, 1, 1, 1, 0.3\} \end{aligned}$$

Now we move towards defuzzification process. According to rule 1 dynamic priority will be very high.

$$\begin{aligned} \mu _{v.high(x)}&= \frac{x-23}{28-23} \qquad \nu _{v.high(x)} = \frac{28-x}{28-22} \\ 0.625&= \frac{x-23}{5} \qquad 0.3 = \frac{28-x}{6} \\ x&= 26.13 \quad \quad \quad x = 26.2 \end{aligned}$$

Now we calculate the crisp value of dynamic priority for process P1 using TS formula. The working of calculating the defuzzified value of very high dp using TS formula is shown in Table 2. The final dynamic priority value for process P1 is $\frac{64.775}{2.45} = 26.44$

**Table 2:** P1: Defuzzification of very high dp using TS formula (data set 1)

x	$\mu _x$	$\nu _x$	$\pi _x$	$X=(1-\pi _x)\mu _x$	$Y=\pi _x\mu _x$	$X+Y$	$x*(X+Y)$
22	0	0.3	0.7	0	0	0	0
23	0	0.3	0.7	0	0	0	0
24	0.2	0.3	0.5	0.1	0.1	0.2	4.8
25	0.4	0.3	0.3	0.28	0.12	0.4	10
26	0.6	0.3	0.1	0.54	0.06	0.6	15.6
27	0.625	0.166	0.208	0.495	0.130	0.625	16.87
28	0.625	0	0.375	0.391	0.234	0.625	17.5
						2.45	64.775

#### Calculating dynamic priority of process P2

The same procedure is followed for process P2 as well. The fuzzified membership and non-membership values of burst time (6) and nice value (7) for process P2 are shown below:

$$\begin{aligned} \mu _\mathrm{{B.T}} &= \{0.25, 0.125, 0\} ; \nu _\mathrm{{B.T}} = \{0.6, 0.7, 1\} \\ \mu _\mathrm{{N.V}} &= \{0.5, 0.07, 0\} ; \nu _\mathrm{{N.V}} = \{0.412, 0.764, 1\} \end{aligned}$$

After intuitionistically fuzzifying the two input parameters the rules of inference engine are triggered. In case of membership rules 1, 2, 4, and 5 are triggered with dynamic priority very high, high, average and average respectively with truth values shown below:

$$\begin{aligned} low \bigwedge low &= 0.25 \bigwedge 0.5 = 0.25 \\ low \bigwedge med &= 0.25 \bigwedge 0.07 = 0.07 \\ med \bigwedge low &= 0.125 \bigwedge 0.5 = 0.125 \\ med \bigwedge med &= 0.125 \bigwedge 0.07 = 0.07 \end{aligned}$$

In case of non-membership also rule 1, 2, 4, and 5 are triggered with dynamic priority very high, high, average and averge respectively with truth values shown below:

$$\begin{aligned} low \bigvee low &= 0.6 \bigvee 0.412 = 0.6 \\ low \bigvee med &= 0.6 \bigvee 0.764 = 0.764 \\ med \bigvee low &= 0.7 \bigvee 0.412 = 0.7 \\ med \bigvee med &= 0.7 \bigvee 0.764 = 0.764 \end{aligned}$$

Finally, the membership and non-membership values of the output variable dynamic priority for process P2 are shown below:

$$\begin{aligned} \mu _\mathrm{{dp}} = \{0, 0, 0.125/.07, 0.07, 0.25\} ; \nu _\mathrm{{dp}} = \{1, 1, 0.7/0.764, 0.764, 0.6\} \end{aligned}$$

Now we move towards defuzzification process. According to rule 1 dynamic priority will be very high.

$$\begin{aligned} \mu _{v.high(x)}&= \frac{x-23}{28-23} \quad \quad \quad \nu _{v.high(x)} = \frac{28-x}{28-22} \\ 0.25&= \frac{x-23}{5}\quad \quad \quad 0.6 = \frac{28-x}{6} \\ x&= 24.25 \quad \quad \quad x = 24.4 \end{aligned}$$

The working of calculating the defuzzified value of very high dp using TS forumla is shown in Table 3.

**Table 3:** P2: Defuzzification of very high dp using TS formula (data set 1)

x	$\mu _x$	$\nu _x$	$\pi _x$	$X=(1-\pi _x)\mu _x$	$Y=\pi _x\mu _x$	$X+Y$	$x*(X+Y)$
22	0	0.6	0.4	0	0	0	0
23	0	0.6	0.4	0	0	0	0
24	0.2	0.6	0.2	0.16	0.04	0.2	4.8
25	0.25	0.5	0.25	0.187	0.0625	0.25	6.25
26	0.25	0.333	0.416	0.415	0.104	0.25	6.5
27	0.25	0.166	0.583	0.104	0.146	0.25	6.75
28	0.25	0	0.75	0.062	0.187	0.25	7
						1.2	31.3

According to rule 2 dynamic priority will be high.

$$\begin{aligned} \mu _{high(x)}&= \frac{x-16}{21-16}\quad \quad \quad \nu _{high(x)} = \frac{21-x}{21-15} \\ 0.07&= \frac{x-16}{5} \quad \quad \quad 0.764 = \frac{21-x}{6} \\ x&= 16.35 \quad \quad \quad x = 16.4 \\ \mu _{high(x)}&= \frac{26-x}{26-21}\quad \quad \quad \nu _{high(x)} = \frac{x-21}{27-21} \\ 0.07&= \frac{26-x}{5} \quad \quad \quad 0.764 = \frac{x-21}{6} \\ x&= 25.65 \quad \quad \quad x = 25.584 \\ \end{aligned}$$

The working of calculating the defuzzified value of high dp using TS forumla is shown in Table 4.

**Table 4:** P2: Defuzzification of high dp using TS formula (data set 1)

x	$\mu _x$	$\nu _x$	$\pi _x$	$X=(1-\pi _x)\mu _x$	$Y=\pi _x\mu _x$	$X+Y$	$x*(X+Y)$
22	0	0.6	0.4	0	0	0	0
23	0	0.6	0.4	0	0	0	0
24	0.2	0.6	0.2	0.16	0.04	0.2	4.8
25	0.25	0.5	0.25	0.187	0.0625	0.25	6.25
26	0.25	0.333	0.416	0.415	0.104	0.25	6.5
27	0.25	0.166	0.583	0.104	0.146	0.25	6.75
28	0.25	0	0.75	0.062	0.187	0.25	7
						1.2	31.3

According to rule 4 dynamic priority will be average.

$$\begin{aligned} \mu _{avg(x)}&= \frac{x-9}{14-9}\qquad \qquad \quad \nu _{avg(x)} = \frac{14-x}{14-8} \\ 0.125&= \frac{x-9}{5}\qquad \qquad \quad 0.7 = \frac{14-x}{6} \\ x&= 9.63\qquad \qquad \quad x = 9.8 \\ \mu _{avg(x)}&= \frac{19-x}{19-14}\qquad \qquad \nu _{avg(x)} = \frac{x-14}{20-14} \\ 0.125&= \frac{19-x}{5}\qquad \qquad \quad 0.7 = \frac{x-14}{6} \\ x&= 18.4\qquad \qquad \quad x = 18.2 \end{aligned}$$

The working of calculating the defuzzified value of average dp using TS forumla is shown in Table 5.

**Table 5:** P2: Defuzzification of average dp using TS formula (data set 1)

x	$\mu _x$	$\nu _x$	$\pi _x$	$X=(1-\pi _x)\mu _x$	$Y=\pi _x\mu _x$	$X+Y$	$x*(X+Y)$
22	0	0.6	0.4	0	0	0	0
23	0	0.6	0.4	0	0	0	0
24	0.2	0.6	0.2	0.16	0.04	0.2	4.8
25	0.25	0.5	0.25	0.187	0.0625	0.25	6.25
26	0.25	0.333	0.416	0.415	0.104	0.25	6.5
27	0.25	0.166	0.583	0.104	0.146	0.25	6.75
28	0.25	0	0.75	0.062	0.187	0.25	7
						1.2	31.3

According to rule 5 dynamic priority will again be average.

$$\begin{aligned} \mu _{avg(x)}&= \frac{x-9}{14-9}\quad \quad \quad \nu _{avg(x)} = \frac{14-x}{14-8} \\ 0.07&= \frac{x-9}{5}\quad \quad \quad 0.764 = \frac{14-x}{6} \\ x&= 9.35\quad \quad \quad x = 9.42 \\ \mu _{avg(x)}&= \frac{19-x}{19-14} \quad \quad \quad \nu _{avg(x)} = \frac{x-14}{20-14} \\ 0.07&= \frac{19-x}{5} \quad \quad \quad 0.764 = \frac{x-14}{6} \\ x&= 18.65\quad \quad \quad x = 18.58 \end{aligned}$$

The working of calculating the defuzzified value of average dp using TS forumla is shown in Table 6.

**Table 6:** P2: Defuzzification of average dp using TS formula (data set 1)

x	$\mu _x$	$\nu _x$	$\pi _x$	$X=(1-\pi _x)\mu _x$	$Y=\pi _x\mu _x$	$X+Y$	$x*(X+Y)$
22	0	0.6	0.4	0	0	0	0
23	0	0.6	0.4	0	0	0	0
24	0.2	0.6	0.2	0.16	0.04	0.2	4.8
25	0.25	0.5	0.25	0.187	0.0625	0.25	6.25
26	0.25	0.333	0.416	0.415	0.104	0.25	6.5
27	0.25	0.166	0.583	0.104	0.146	0.25	6.75
28	0.25	0	0.75	0.062	0.187	0.25	7
						1.2	31.3

The final dynamic priority value for process P2 is obtained by averaging out all the four dynamic priorities computed above, i.e., $\frac{26.08 + 20.5 + 14 + 14}{4} = 18.65$.

The same procedure is followed for computing the dynamic priorities for the rest of the processes in the data set. Finally, the dynamic priority for processes P1, P2, P3, P4 and P5 comes out to be 26.44, 18.65, 26.33, 26.2, and 26.53 respectively.

#### Comparison

If we sort the processes according to the calculated dynamic priorities, the sequence of execution of the processes is $\left\langle P5, P1, P3, P4, P2 \right\rangle$. The average waiting time for this data set comes out to be 6 time units. The same results are obtained if we follow the process with the smallest burst time, i.e., shortest job first algorithm or if we simply execute the processes according to their nice value. The process with smaller nice value is selected for execution. Table 7 shows the results of data set-1. If these results are compared with author’s previous paper (Butt and Akram 2015), it can be observed that with a time slice of 6 units the results are the same as Shortest Job First, which is a scheduling algorithm for batch operating systems. Intuitively, the given data set is such that the same results are produced as both the parameters are not conflicting. However, it is quite intuitive to observe that if the burst time and nice value of processes in the data set are conflicting to each other our algorithm will give better results than the SJF and the priority based algorithm. This is shown in the second data set.

**Table 7:** Results: data set-1

Algorithm	Average waiting time
Shortest job first	6
Priority based	6
IF scheduler	6

#### Sensitivity analysis

According to Rezaei and Ortt (2013), sensitivity analysis is used to determine how sensitive the output of a system is on a particular input. Sensitivity analysis is useful as it can be used to determine how dependent the output is on a specific input. The formula to compute the contribution of an input on the output as given in Rezaei and Ortt (2013) is shown below:

$$\begin{aligned} \Delta _{i} = \frac{ \sum _{j=1}^m |dp^{'}(j) - dp(j)|}{m} \end{aligned}$$

In the above formula *dp*(*j*) is the dynamic priority of a process that is computed considering all available inputs. $dp^{\prime}(j)$ is the dynamic priority of a particular process by removing all information of the *i*th input from the rule base. $\Delta _{i}$ shows the average absolute change after removing $i^{th}$ input. In our proposed intuitionistic fuzzy system, there are only two inputs, nice value (NV) and burst time (BT). For process P1 (BT:3, NV:2) and P2 (BT:6, NV: 7) the defuzzified dynamic priorities (*dp*) co-latexmputed above are 26.44 and 18.65 respectively. If we drop all the information regarding nice value (NV) for both processes, the new dynamic priorities ($dp^{\prime}$) are 26.438 and 19.792 respectively. Applying sensitive analysis formula $\Delta _{NV} = \frac{ |(26.438 - 26.44)| + |(19.792 - 18.65)|}{2}$, which comes out to be 0.572. Similarly, $\Delta _{BT} = \frac{ |(26.1428 - 26.44)| + |(20.1665 - 18.65)|}{2}$ is computed as 0.9068. This shows that in data set 1, shown in Table 1, burst time (BT) has more important contribution in the final output of the system, while nice value (NV) has lesser contribution.

### Data set 2

The input for data set-2 is given in Table 8. These per process values are taken from author’s previous paper (Butt and Akram 2015). The recent cpu usage of each process is not computed here, as this input is not required for scheduling algorithms of batch operating systems. The first column shows the five processes under consideration, with their burst times (BT) and nice values (NV) shown against them.

**Table 8:** Data set-2

PID	BT	NV
P1	20	5
P2	4	31
P3	18	7
P4	6	25

Table 8 shows that there are four processes with different burst times and nice values. We need to compute the dynamic priority of all the four processes. The detailed working for computing the dynamic priority of these processes is not shown as the same is shown in the previous case study. The same procedure is followed for computing the dynamic priorities of processes in data set 2. Finally, the dynamic priority for processes P1, P2, P3, and P4 comes out to be 10.5, 20.9, 14, and 17.5 respectively.

#### Comparison

If we sort the processes according to the calculated dynamic priorities, the sequence of execution of the processes is $\left\langle P2, P4, P3, P1 \right\rangle$. The average waiting time for this data set comes out to be 10.5 time units. The same results are obtained if we follow the process with the smallest burst time, i.e., shortest job first algorithm. If we simply execute the processes according to their nice value i.e., the process with smaller nice value is selected for execution, the waiting time comes out to be 25.5 time units. Table 9 shows the results for data set-2.

**Table 9:** Results: data set-2

Algorithm	Average waiting time
Shortest job first	10.5
Priority based	25.5
IF scheduler	10.5

## Conclusion and future work

We have extended our previous algorithm, fuzzy decision making system for CPU scheduler of a multi-tasking OS (Butt and Akram 2015), to intuitionistic fuzzy (IF) modeling techniques. Our proposed algorithm has shown results that are comparable to the best-known non-preemptive scheduler, i.e., shortest job first (SJF). We have presented a novel decision-making system based on intuitionistic fuzzy sets for CPU scheduling algorithm of a batch operating system. The main limitation of this algorithm is its complexity which is not a major concern in case of a batch operating system. The same can be extended for multi-tasking operating systems where complexity of scheduling algorithm is not a concern. Improving the selection criteria of a CPU scheduling algorithm is a hot area of research. Our proposed algorithm can give even better results if it can identify between batch and interactive processes, and intelligently increase/decrease the niceness of batch/interactive processes respectively. To identify between a batch and interactive process, one can compute and keep record of the average sleep time spent by every process in its lifetime. This will improve the overall inter-activeness of a computer system to manifold.
